# Design and Implementation of a Comprehensive AI Dashboard for Real-Time Prediction of Adverse Prognosis of ED Patients

**DOI:** 10.3390/healthcare10081498

**Published:** 2022-08-09

**Authors:** Wei-Chun Tsai, Chung-Feng Liu, Hung-Jung Lin, Chien-Chin Hsu, Yu-Shan Ma, Chia-Jung Chen, Chien-Cheng Huang, Chia-Chun Chen

**Affiliations:** 1 Department of Emergency Medicine, Chi Mei Medical Center, Tainan 71004, Taiwan; 2 Department of Pediatrics, Chi Mei Medical Center, Tainan 71004, Taiwan; 3 Department of Public Health, College of Medicine, National Cheng Kung University, Tainan 70101, Taiwan; 4 Department of Medical Research, Chi Mei Medical Center, Tainan 71004, Taiwan; 5 Department of Information Systems, Chi Mei Medical Center, Tainan 71004, Taiwan; 6 Department of Emergency Medicine, Kaohsiung Medical University, Kaohsiung 80708, Taiwan; 7 Department of Environmental and Occupational Health, College of Medicine, National Cheng Kung University, Tainan 70101, Taiwan; 8 Department of Information Systems, Chi Mei Medical Center, Liouying 73657, Taiwan

**Keywords:** artificial intelligence, machine learning, big data, emergency department, dashboard, prognosis, prediction model

## Abstract

The emergency department (ED) is at the forefront of medical care, and the medical team needs to make outright judgments and treatment decisions under time constraints. Thus, knowing how to make personalized and precise predictions is a very challenging task. With the advancement of artificial intelligence (AI) technology, Chi Mei Medical Center (CMMC) adopted AI, the Internet of Things (IoT), and interaction technologies to establish diverse prognosis prediction models for eight diseases based on the ED electronic medical records of three branch hospitals. CMMC integrated these predictive models to form a digital AI dashboard, showing the risk status of all ED patients diagnosed with any of these eight diseases. This study first explored the methodology of CMMC’s AI development and proposed a four-tier AI dashboard architecture for ED implementation. The AI dashboard’s ease of use, usefulness, and acceptance was also strongly affirmed by the ED medical staff. The ED AI dashboard is an effective tool in the implementation of real-time risk monitoring of patients in the ED and could improve the quality of care as a part of best practice. Based on the results of this study, it is suggested that healthcare institutions thoughtfully consider tailoring their ED dashboard designs to adapt to their unique workflows and environments.

## 1. Introduction

The busiest department and most flocked area in the hospital is the emergency room (ED), which serves as the forefront of medical care. Patients come to ED for acute illness and critical conditions. Quick and correct patient evaluation and feedback on medical advice are important. The medical team is required to make treatment decisions under huge pressure and in a short amount of time. Physicians and nurses assigned to ED are usually stressed because they need to attend to patients with diverse medical conditions, which, oftentimes, change rapidly. It is therefore vital to make real-time, personalized, and accurate disease predictions in the ED. Unfortunately, the support of the hospital information system (HIS) for clinical decision-making is still insufficient [1]. Although many ED decision support systems (DSS) have been proposed [2,3,4,5], they are mostly research-oriented and have not been adopted for clinical use.

Artificial intelligence (AI) in healthcare is becoming increasingly essential due to its potential to enhance clinical risk predictions and advanced administration within the organization. Thus, implementing AI to achieve these desired benefits is expected in ED [6]. Therefore, focusing on practicalities, the ED in Chi Mei Medical Center (CMMC) has developed and implemented eight artificial intelligence (AI) disease predictive systems and integrated them into the HIS, including influenza in old age, chest pain, pancreatitis, hyperglycemic crisis, dengue, pneumonia, brain trauma, and fever. These predictive systems have been investigated and implemented, and the relevant results were published [7,8,9,10].

However, past research claims that the current medical healthcare AI available is only used to assist in medical decision-making. Thus, our hospitals adopted a spontaneous use, without compulsion, and the AI predictive results were provided for reference [11]. As a result, the ED healthcare staff did not use the medical AI tools often. Therefore, it is crucial to determine how medical AI can be transformed from its current passive role to an active form of support. The use of an AI dashboard to actively monitor patients’ conditions is one of the most feasible ways to utilize it, as has been realized in the respiratory care center (RCC) of CMMC [12]. The dashboard in the RCC clearly shows the success probability of weaning from the ventilator for each intubated patient, with an excellent reference for decision-making. It was also found that the average intubation time indeed decreased after AI intervention.

In light of the early successful experience in the RCC, we integrated the AI outcome prediction models for the eight types of disease diagnosis in the ED. This was achieved in the form of a dashboard that monitors the prognostic risk of the patients currently in the ED. Therefore, the purpose of this study is to systematically share the successful experience of CMMC in developing this ED AI dashboard, and to serve as an important reference for the development of AI in other hospitals by providing the overall AI infrastructure and software operation mode.

## 2. Materials and Methods

### 2.1. The Pathway of AI Development in CMMC

Under the instructions of the board of directors and the president, CMMC officially established the Center for Big Medical Data and AI Computing (AI Center) in May 2019 as the base for medical AI development for the three hospitals of the Chi Mei Medical Group (CMMG). The AI Center has three main goals: (1) to establish a medical big data database (data warehouse); (2) to promote AI research; and (3) to develop AI applications. A data scientist and professor hosts the AI Center and recruits data analysis experts and information management experts. In addition, the Department of Information Systems serves as a critical external support for the establishment of AI infrastructure, including the establishment of a big data server, graphical processing unit (GPU) server, and web service (WS) server. After its establishment, CMMG actively encouraged various medical departments in Chi Mei hospitals to develop AI based on big data from EMR (electronic medical records). As of June 2022, more than 20 AI models for prognosis prediction have been developed and implemented in clinical practice.

### 2.2. The AI Computing Infrastructure in CMMC

**Figure 1:**
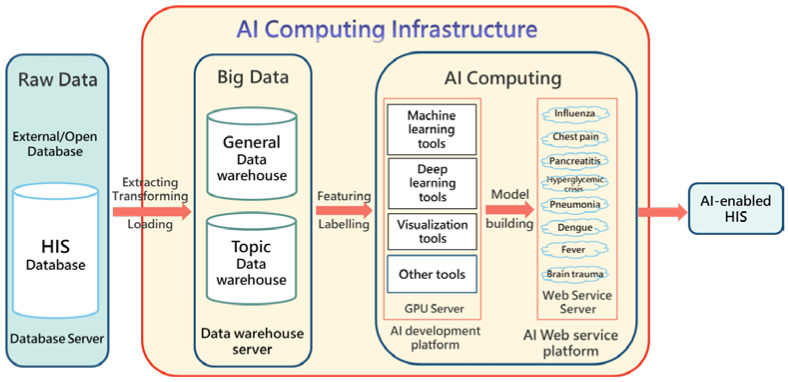
AI computing infrastructure of CMMC (ED as an example).

Chi Mei’s AI computing infrastructure (yellow area in Figure 1) consists of three cores, namely, medical big data, an AI development platform (including GPU), and an AI web service platform [11]. The original data source (data warehouse) is mainly the online hospital information system (HIS) database (historical EMR). Data from the HIS are routinely transferred to the big data system through the ETL procedures (extracting, transferring, and loading using a professional tool called IBM InfoSphere DataStage^®^). The big data are then used as a data source for machine learning and other retrospective studies. The design purposes of the database and data warehouse are completely different. The former generally refers to any collection of data organized for storage, accessibility, and retrieval, while the latter is a special database that replicates and integrates the data of the operation process (e.g., EMR) to facilitate statistics and analysis. Chi Mei builds two levels of big data; one is a general-purpose data warehouse, that is, the data used in most ML research, such as lab data and physicians’ orders; the other is a specific-purpose data warehouse, designed for specific fields of ML studies, such as stroke and cancer. The emerging artificial intelligence of things (AIoT) combines AI and the Internet of things (IoT) and involves the integration of sensing technologies to solve real-world problems [13]. We also adopted AioT to retrieve the vital signs of the patients from their medical instruments and perform AI predictions accordingly.

Machine learning often incorporates a huge amount of data (e.g., tens of thousands); thus, it is necessary to build a GPU server to improve the computing efficiency. In addition, even though there are many free machine learning development platforms (e.g., Anaconda platform), we adopted a professional ML development platform (InfuseAI PrimeHub^®^) in order to effectively manage multiple users, perform modeling, allocate computing resources, distribute models, and manage versions.

Finally, we developed all the ED AI models into various web services (AI web service, AWS) to form a model bank, which was then placed on a web service platform. As long as the existing HIS sends the necessary feature values (e.g., age, sex, weight, creatinine, etc.) required by a specific AI model to the web service platform through the URL connection, the associated AWS will return the prediction result (e.g., a 0.23 probability of death).

The raw data include the existing HIS database and external/open database (data imported from outside or not generated by the hospital itself, such as ICD-10 code data and the national health insurance code data). After integrating it into the AI web services, the HIS is equipped with AI-assisted functions, which can be called AI-enabled HIS.

### 2.3. Software Operation Mode

**Figure 2:**
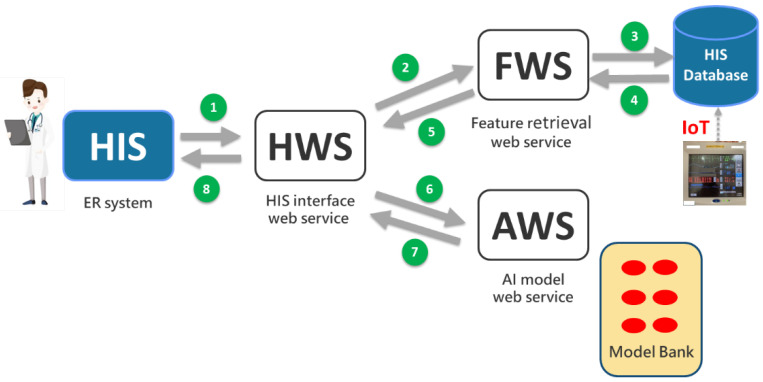
Web service operation mode. Note. The sequence of interactive steps of WS is shown in numerical order in the figure.

We used service-oriented architecture (SOA) [14] to build the overall software operation and to design three kinds of web services: the HIS interface web service (HWS), the feature retrieval web service (FWS), and the AI model web service (AWS). The operational steps of the WSs can be traced using the number order in Figure 2.

- HWS

The HWS receives the calls from the existing ED system and returns the prediction results to the ED system. The HWS also provides the interactive functions allowing physicians to adjust feature values to re-predict and simulate the possible changes in the prognosis.

- FWS

The FWS receives the calls from the HWS to capture the patients’ feature values (such as sex, age, Glasgow Coma Scale, etc.) which are then returned to the HWS. The FWS can also use IoT technology to extract patients’ bedside physiological information (e.g., blood pressure, heart rate, etc.).

- AWS

We developed all the AI prediction models into AI WSs (AWS) and stored them in the Model Bank. The Model Bank is installed on the AI Web Service Platform to await connection calls from the HIS. The AWS receives the calls of the HWS, obtains the patients’ feature values, and returns the results of the AI prediction (probability value) to HWS. For example, the AWS of ED chest pain returns the risk probability of acute myocardial infarction to the HWS.

### 2.4. AI Prognostic Prediction Model Development

**Figure 3:**
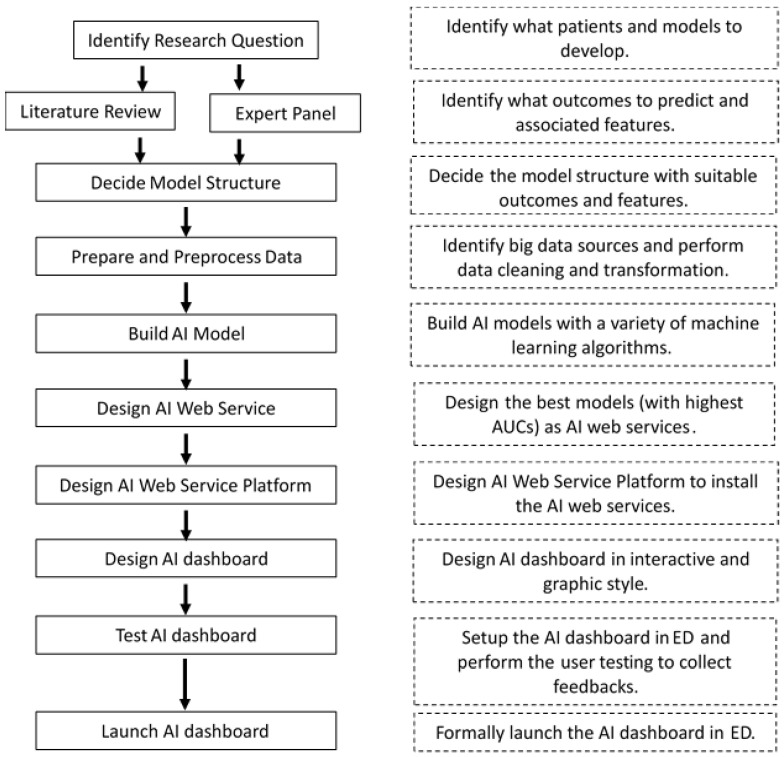
Steps of the model building and dashboard implementation. Note: AUC, the area under the ROC curve.

Based on the huge EMR, we successively developed eight prognostic prediction systems (in WS form) in the ED. The steps of the model building and implementation are shown in Figure 3.

We developed several outcome prediction models for each type of patient (such as sepsis models, death models, etc.), and each prediction model was built with multiple machine learning and deep learning algorithms (such as Multilayer Perceptron, Random Forest, XGBoost, and others). For example, we simultaneously used five algorithms to build the prediction model for death due to influenza in the ED among the elderly (i.e., five models were built for a single outcome prediction). We then compared the quality of the five models built to determine which was the best one. Usually, the one with the highest AUC (the area under the ROC or receiver operator characteristic curve) is the best model, but the balance between sensibility and specificity still needs to be considered.

Our feature selection strategy for each model was based on the following considerations: (1) relevant medical literature and clinical experience; (2) statistical significance (T-test or Chi-square test); (3) correlation (Spearman test or Pearson test); and (4) feature importance graphs. Currently, we have only developed AI prediction systems based on structured electronic medical records (non-image data). The values of the patient parameters selected were directly extracted from the HIS, which were then sent to the model prediction software (called the AWS in this study), and the predicted results were returned to the system. To ensure feasibility, we did not perform complex feature extraction and fusion in the data preprocessing (e.g., no PCA techniques were utilized) in order to develop and integrate the AI software more modularly and easily. Again, to ensure feasibility, the number of features selected was generally limited to under 30.

### 2.5. AI Dashboard Architecture

Because the ED is generally very busy, the medical staff do not often have time to use AI prediction functions. As a result, we considered encouraging the staff to actively engage in the AI system by developing a platform that helps to predict every patient’s condition automatically while working in the background. This platform allows another group of medical staff to monitor patient conditions and give timely responses if necessary. Thus, the AI dashboard design was proposed.

Based on the principles and guidelines for the design and technical architecture of enterprise dashboards in health care [15], we proposed a four-tier system architecture for our ED dashboard, which are: (1) the presentation layer, (2) the application layer, (3) the model layer, and (4) the infrastructure layer.

#### 2.5.1. Presentation Layer

The presentation layer focuses on the use and display of the operation interface. Easy-to-use and graphical representations were key to its design.

- Real-time and automated prediction:

All patients are instantly scanned in the emergency room. As long as they meet the classification of the eight types of diseases, the feature values required by the model from the HIS database are automatically captured and the probability of prediction (risk) is calculated in an instant. Individuals working in the emergency room are required to have a great sense of urgency, so they experience extreme pressure. It would be unsuitable to require health practitioners in the ED to input the feature values to obtain the predicted results. Only real-time and automatic prediction can be accepted in this type of clinical practice.

For example, when a patient has just been admitted to the ED and is coded as elderly with influenza by the physician (ICD9 codes as 487 or 488), the dashboard will automatically capture the patients’ 10 feature values of influenza in the elderly models (tachypnea, GCS, history of hypertension, history of CAD, history of malignancy, bedridden, leukocytosis, bacteremia, anemia, and elevated CRP) from the HIS to calculate the risk probabilities of the five outcomes (hospitalization, pneumonia, sepsis or septic shock, ICU admission, and in-hospital death) [8].

Moreover, the AI dashboard can capture the patients’ vital signs in a timely manner using IoT technology to perform the prediction and update the prediction results at regular intervals (currently set to refresh once every 10 min).

- Graphical interface presentation:

The prediction results are presented directly with a concise circular figure (risk value), which allows the user to view the result at a glance, helping one to respond quickly. In addition, the AI dashboard displays each feature value for reference. The results of each prediction are then kept in a line chart showing changes in the patients’ continuous risk.

- Interactive simulation prediction:

The developed dashboard provides interactive functions, allowing users to manually adjust the feature values to simulate possible changes in risk. This helps the medical team to plan the more appropriate treatment for possible changes in condition. The interactive function is also a useful SDM (shared decision-making) tool. Through presenting scientific digits and figures, it becomes a convenient tool for healthcare staff in explaining the patient condition to the patients or their family members.

#### 2.5.2. Application Layer

We positioned the AI dashboard as a medical assistant tool used to monitor the risks of the patients in a timely manner and to simulate possible changes in outcome through the interactive functions, allowing healthcare providers to respond in time. Currently, the AI dashboard can provide risk predictions for patients diagnosed with any of the eight diseases mentioned. In the future, other features and applications of the AI dashboard can be added, such as the monitoring of patients’ waiting time for treatment.

#### 2.5.3. Model Layer

The Model Layer reveals the source of the AI dashboard’s excellent capabilities; that is, it combines all the available AI models to provide various condition prediction functions. These models are physically stored in the Model Bank. At present, eight models for the eight patient conditions, including influenza in old age, chest pain, pancreatitis, hyperglycemic crisis, dengue, pneumonia, brain trauma, and fever are available in the model layer.

#### 2.5.4. Infrastructure Layer

- AI web service platform:

The AI platform is a container-enabled computer server (Figure 1), consisting of the Model Bank (where a variety of ED prediction models are stored) and web service control mechanism (Figure 2). It receives the connection request from an existing HIS at any time and returns the prediction results. A Docker container image is used in the AI platform. It is a lightweight, standalone, and executable package of software that includes everything needed to run an application, such as our AI web service.

- Related development tools:

In order to meet the needs of the various specifications of AI models, we used JSON (JavaScript Object Notation) as the exchange format between the web services. JSON is an open-state format and data interchange format, including that of web applications with servers. Both the web services and the web service platform were developed in Microsoft Visual Studio.Net^®^ to ensure that they were compatible with the existing HIS. The AI prediction models were developed on the Jupyter Notebook platform. In response to the model algorithms and analysis functions, the development platform has to be installed with the corresponding open libraries, such as TensorFlow, Scikit-Learn, and so on.

## 3. Results

### 3.1. AI Models Developed in the Emergency Department

Since 2019, the CMMC ED has developed the AI disease prediction models for the eight conditions (influenza, chest pain, pneumonia, pancreatitis, hyperglycemia, dengue fever, brain trauma, and fever) based on the big data recorded over the past ten years. Each type of patient has several outcome prediction models. For instance, the AI for chest pain has two prediction models (AMI, death) [10]; the AI for influenza in the elderly has five prediction models (hospitalization, pneumonia, sepsis/septic shock, ICU admission, and death) [8]; the AI for pneumonia has three prediction models (respiratory failure, sepsis/septic shock, and death) [9]; and the AI for brain trauma has three prediction models (ICU admission, hospitalization, and death) [7]. All the AI prediction models and their launch times are shown in Table 1.

### 3.2. The AI Dashboard

**Figure 4:**
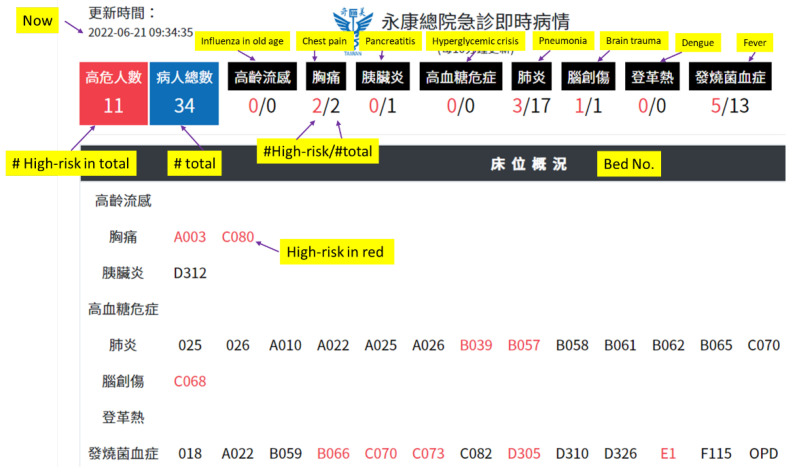
AI dashboard portal.

**Figure 5:**
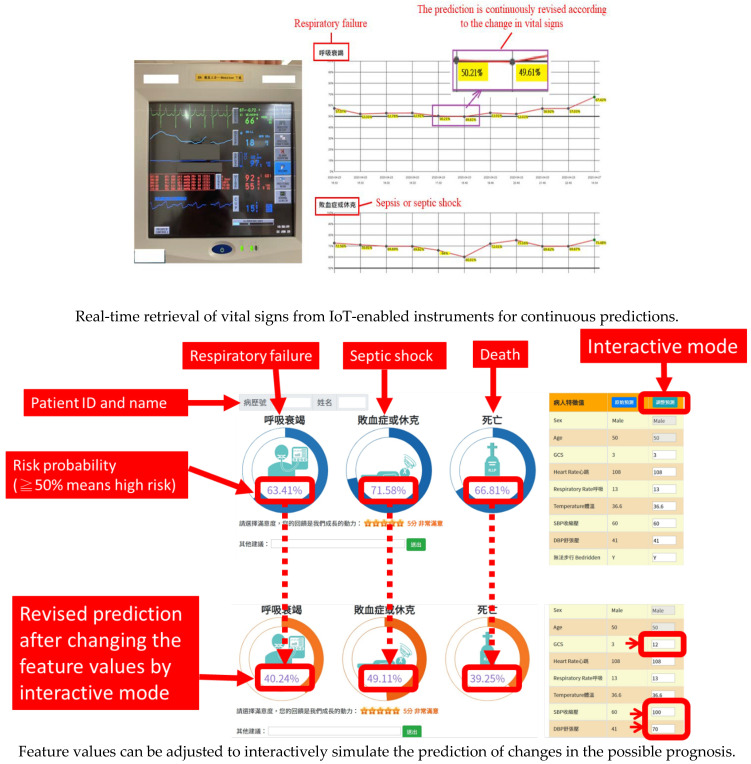
Features of IoT-enabled and interactive functions (pneumonia patient). Note: IoT, Internet of things.

Figure 4 shows the general picture of the AI dashboard currently being implemented in the ED. It displays the current state of all patients with any of the eight diseases in the ED and shows the degree of risk in a highly visible text and color. The bed numbers in red denote high-risk patients (for example, the pneumonia patient in B039 is high risk). Users can click the bed to browse through the detailed results (Figure 5). High risk, in red, means that at least one of the outcome risk probabilities is equal to or above 50%.

### 3.3. Clinical Adoption in Three Hospitals

**Figure 6:**
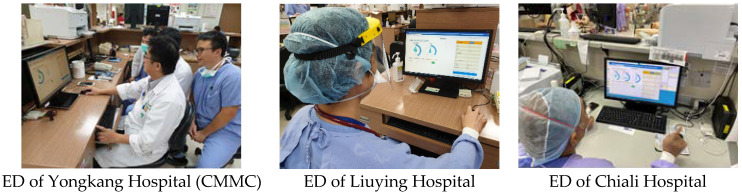
The EDs of the three branch hospitals have launched the AI dashboard.

CMMG’s HIS was fully developed by its own Department of Information Systems (with a few exceptions, such as PACS). After the AI dashboard test was completed, it was launched simultaneously in the three hospitals. As shown in Figure 6, the AI dashboard is installed on each PC of the work station, and any of the medical staff can use it. The main users of the dashboard are emergency supervisors, chief physicians on duty, and the ED head nurse. They can monitor the status of patients diagnosed with any of the eight conditions through the AI dashboard in a timely manner, pay special attention to the high-risk ones, and provide instant support or treatments if necessary (especially if the patient’s attending physician is serving other patients).

### 3.4. User Acceptance Evaluation

After a month of pilot use, 10 ED staff (7 physicians and 3 nurses) completed the acceptance investigation. They were asked to answer a five-point questionnaire based on the concept of the Technology Acceptance Model (TAM) [16]. Their average score was above 4.2 in regard to perceived ease of use, perceived usefulness, and perceived acceptance, which indicates high satisfaction with, and high intention to use, the ED dashboard.

In terms of ease of use, the respondents felt that the ED dashboard was very useful because the graphical interface is clear at a glance and the prediction does not require artificial input parameters. In terms of usefulness, since the AI dashboard can continue to assess the patients’ risk level, it permits the medical team to respond to the needs of the patients promptly, helping to improve the safety of the patients. In general, the metrics of the model performance were almost above 0.7. The physicians expressed that they could accept the AI dashboard as a tool to assist and support their clinical decisions. The respondents mentioned that they were very much willing to use this in the ED. However, they also admitted that specific benefits still require long-term observation and verification.

### 3.5. Preliminary Impact Analysis after AI Assistance

The AI dashboard was integrated into the existing emergency system to assist physicians with their decision-making. However, physicians still had a choice and could decide whether to use AI or not (i.e., to click the prediction button). Since CMMC only encouraged physicians to use AI rather than making it mandatory, we were able to observe the differences in the outcomes of patients whose physicians used the AI prediction and those who did not. We performed a comparison based on a preliminary impact analysis after the AI was launched and implemented in the three hospitals. We found that patients treated using the AI predictions by physicians seemed to have better outcomes than patients treated without AI predictions (Table 2). This indicates that the AI intervention may have a positive effect on the patients.

## 4. Discussion

We proposed and implemented a four-tier AI dashboard architecture for ED clinical use. We also introduced the overall AI infrastructure and outlined the steps for developing the AI prediction model with an easy-to-access EMR that can be obtained by hospitals. The concept of an AI dashboard can enable healthcare AI to actively evaluate all the patients at the ED (the number of annual patient visits at the ED in CMMC is around 100,000) and can enhance the utility of AI. To our best knowledge, the present study is the first research investigation on medical informatics that designed and implemented an AI dashboard in the ED. Some previous studies had a patient-oriented dashboard implemented in the ED [17,18]. This kind of traditional dashboard focused on simplifying the ED patient flow and notifying clinicians and nurses of the patients’ summarized data. Our study presented an AI dashboard that actively supervised ED patients diagnosed with any of the eight conditions mentioned in real-time. On the dashboard, the bed numbers of high-risk patients are displayed in red color for better visibility, which can be easily seen even at a glance. Other studies provided individual predictions for specific diseases or patients using AI systems [19,20]. However, our AI dashboard empowers individual prediction, not only for the newly admitted patients but for all patients currently in the ED. Table 3 presents a comparison of this study with related studies based at EDs.

In accordance with the principles and guidelines obtained in this study, we pointed out how the infrastructure of a data warehouse, an AI development platform with GPU computing power, and an AI web service platform can be constructed for the effective delivery of AI predictions. The web service interactive mode we proposed provides a flexible and modular software design approach, which is conducive to the rapid expansion and migration of the AI dashboard. Therefore, the presentation of all valuable risk values of the patients along with interaction using graphical styles made the ED dashboard highly accepted by healthcare workers.

This AI dashboard integrated eight healthcare AI systems and actively evaluated and notified the healthcare providers in real time about patients with a relatively higher risk of adverse outcomes. It can be used during duty briefing and then monitoring, as needed, by nurse practitioners at the emergency observation area. In this way, more healthcare AI applications for better quality care in the ED could be implemented. When a higher-risk patient is shown in red color on the AI dashboard, indicating that the AI criteria were met and that the patient has at least one adverse outcome risk higher than 50 percent, the nurse leader can visit this patient and remind the doctor of the patient’s evaluation and management. For example, one of our AI models is a chest pain AI system. If the acute myocardial infarction predictive rate is very high, real-time information can notify the primary doctor to closely monitor this patient and consult the cardiologist if the clinical prediction favors acute myocardial infarction. Another example is our pneumonia AI system. Continuous predictions of respiratory failure can be obtained because the AI model can actively gather a patient’s oxygen saturation, heart rate, and respiratory rate through a monitor. This real-time information can assist the primary doctor in re-evaluating the patient’s clinical response to treatment. Furthermore, the feature variables, except for the patient’s age and sex, can be adjusted by healthcare providers. For instance, after the initial management, when the patient’s chest pain and troponin–I improve, the primary nurses can refresh the AI model or adjust the new value of troponin–I manually. This will prompt the AI model to show a better predictive rate of acute myocardial infarction.

Using an AI dashboard can make the work of the ED more efficient. The AI dashboard can quickly update the evaluation of the patients in the HIS every ten minutes and actively classify and organize high-risk patients and non high-risk patients by the font and color of their bed number (high-risk patients in red, non high-risk in black). The AI systems in the dashboard include those for influenza in old age, chest pain, pancreatitis, hyperglycemic crisis, dengue, pneumonia, brain trauma, and fever. These eight diseases were chosen for the development of AI models by our ED physicians because patients diagnosed with these diseases are usually in critical conditions. After implementing these AI systems in the ED for 2 years, the mortality rates and the ICU transposition rates of influenza in old age, chest pain, pancreatitis, hyperglycemic crisis, and pneumonia were found to be lower when the AI predictive systems were used. Since the AI dashboard is user-friendly and utilizes graphics so that it can be easily understood, it can be employed by the physician as a tool for shared decision-making, by showing the graphical representations made by the AI to the patients or their families. So far, AI implementation in the ED is still scarce all over the world [21].

## 5. Conclusions

This study proposed and implemented a four-tier AI dashboard architecture in the ED for clinical use. The results of our study mark an important milestone in the development of a medical AI dashboard. In addition, this study also proposed an overall AI infrastructure and outlined the steps for developing the AI prediction model with an easy-to-access EMR in hospitals. We provided clear guidance for small- and medium-sized hospitals that intend to develop AI applications but do not know where to start. Therefore, this study is deeply aligned with academic and practical contributions.

There were some limitations of the study. Firstly, all of the big data of ED patients were gathered from three branch hospitals of CMMC. Thus, further external validation of the AI models in a diverse hospital is warranted. Moreover, future studies should encourage more hospitals to cooperate so that the study’s generalizability can be extended through federated learning [22] or other methods of merging more hospitals’ big data to improve the quality and stability of the AI models. 

The current AI applications developed by CMMC were mainly based on structuralized electronic medical records. Medical image AI and unstructured EMR AI are important areas and need to be studied and implemented in the future, regardless of whether this concerns individual predictions or group predictions in the dashboard form. Moreover, we call for more AI models for various diseases in the ED to be developed to maximize the coverage of monitored patients. In addition, discussing the success of medical AI at the the environmental and organizational level is a very worthy research topic, including the topics of policy trend driving, value-added and benefit assessment, harmony between stakeholders, and the consensus of the organization [11,23].

Currently, we are planning to develop the AI cloud service platform and share our ED AI services with medical institutions in remote villages and outlying islands. For example, the physicians in a rural hospital can send their ED chest pain patients’ feature variable values through the Internet to the CMMC AI service platform, and the platform can then return the real-time risk predictions. In so doing, the medical team of a tertiary-level hospital can provide assistance and high-quality support to hospitals lacking resources anytime and anywhere. Through AI, the medical digital gaps will be filled.

## Figures and Tables

**Table 1:** The prediction models of each category of patient.

Patient Category	Number of Prediction Model	Prediction Model Name	Accuracy	Sensitivity	Specificity	AUC	Introduction Date
Influenza in old age	5	Hospitalization	0.769	0.744	0.791	0.840	2019.05
		Pneumonia	0.679	0.681	0.679	0.765	
		Sepsis or septic shock	0.795	0.750	0.798	0.857	
		ICU admission	0.912	0.722	0.914	0.902	
		Death	0.816	0.806	0.816	0.889	
Chest pain	2	Acute myocardial infarction	0.850	0.850	0.850	0.923	2019.07
		Death	0.712	0.703	0.712	0.761	
Pancreatitis	3	Sepsis or septic shock	0.737	0.762	0.732	0.801	2019.09
		ICU admission	0.778	0.782	0.777	0.859	
		Death	0.776	0.742	0.777	0.821	
Hyperglycemic crisis	3	Sepsis or septic shock	0.748	0.830	0.716	0.842	2019.11
		ICU admission	0.709	0.735	0.706	0.771	
		Death	0.738	0.752	0.736	0.807	
Dengue	3	Sepsis or septic shock	0.713	0.741	0.706	0.794	2020.03
		ICU admission	0.832	0.941	0.831	0.923	
		Death	0.852	0.933	0.851	0.954	
Pneumonia	3	Respiratory failure	0.728	0.810	0.714	0.847	2020.03
		Sepsis or septic shock	0.708	0.711	0.707	0.781	
		Death	0.728	0.770	0.723	0.835	
Brain trauma	3	ICU admission	0.729	0.760	0.724	0.817	2020.09
		Hospitalization	0.699	0.700	0.698	0.764	
		Death	0.893	0.812	0.894	0.925	
Fever	4	Bacteremia	0.702	0.717	0.700	0.761	2022.02
		Sepsis or septic shock	0.623	0.719	0.604	0.735	
		ICU admission	0.707	0.604	0.733	0.755	
		Death	0.756	0.755	0.756	0.848	
AI dashboard							2021.08

**Table 2:** Comparison of the outcomes of patients whose physicians chose to use the AI prediction vs. those who did not.

Patient Type (Comparison Duration)	Did Not Refer to AI Prediction (n)	Referred to AI Prediction (n)	Outcome	Effect
Pneumonia (2020.4~2020.9)	966	135	Respiratory failure	decreased by 1.87%.
			Sepsis or septic shock	decreased by 9.31%
			Death	decreased by 1.73%
Influenza in old age (2019.6~2021.4)	317	438	ICU admission	decreased by 0.772%
			Death	decreased by 0.772%
Hyperglycemic crisis (2019.12~2021.4)	253	18	ICU admission	decreased by 8.65%
			Death	decreased by 3.91%
Chest pain (2019.8~2021.4)	9619	647	Acute myocardial infarction	decreased by 0.02%
			Death	decreased by 0.03%
Pancreatitis (2019.10~2021.4)	993	120	Sepsis or septic shock	decreased by 0.95%
			Death	decreased by 1.79%
Brain trauma (2020.10~2021.4)	1366	81	ICU admission	decreased by 4.41%

Note: (1) The data obtained during the COVID-19 pandemic were not incorporated for modeling; thus, we only compared the AI intervention data from before 2021. (2) The outcomes of conditions, in very few cases, were not compared; e.g., cases of Dengue were too few to compare, and the fever AI was only launched in 2022, so those data were not also compared.

**Table 3:** Comparison with related studies based at EDs.

Study	Current Study	[17]	[18]	[19]	[20]
Study place	Taiwan	USA	Korea	USA	Germany
Study population	ED patients	ED patients	ED patients	ED patients	ED patients
Predicted outcome	High-risk adverse and critical care events (including hospitalization, sepsis or septic shock, ICU admission, in-hospital mortality, etc.)	Visualization of patients’ summarized data and flow	Visualization of patients’ summarized data and flow	Identification of altered mental status (AMS)	Suggested Diagnoses
AI/ML approach	🗸	N/A	N/A	🗸	🗸
Implementation	🗸	🗸	🗸	🗸	🗸
Real-time and individualized monitoring	🗸	🗸	🗸	N/A	N/A
Digital dashboard	🗸	🗸	🗸	N/A	N/A
AI/ML algorithm	🗸 (including Random Forest, LightGBM, logistic regression, XGBoost, and MLP)	N/A	N/A	🗸 (including NLP, convolutional neural network)	🗸 (No details reported)
Can adjust values to repeat predict	🗸	N/A	N/A	N/A	N/A
Notification alert	🗸	🗸	🗸	N/A	N/A
feature variable	12–30 variables (including patients’ age, sex, Glasgow Coma Scale, vital signs, laboratory data, comorbidities, etc.)	N/A	N/A	Text variable (clinical notes)	Limited variables (including patient demographics, patient history, and information about current complaints)
Testing performance	AUC: 0.735–0.925	N/A	N/A	AUC: 0.985	Accuracy: (0.70–0.85)
Year	2022	2017	2018	2019	2021

Note: AUC, the area under the ROC curve.

## Data Availability

Not applicable.
